# Effects of various seed priming on morphological, physiological, and biochemical traits of rice under chilling stress

**DOI:** 10.3389/fpls.2023.1146285

**Published:** 2023-03-13

**Authors:** Hua Zhang, Xiaoli Zhang, Guoqing Gao, Izhar Ali, Xiaoyan Wu, Maoyan Tang, Lei Chen, Ligeng Jiang, Tianfeng Liang

**Affiliations:** ^1^ Key Laboratory of Crop Cultivation and Physiology, Education Department of Guangxi Zhuang Autonomous Region, Guangxi University, Nanning, China; ^2^ Rice Research Institute, Guangxi Academy of Agricultural Sciences/Guangxi Key Laboratory of Rice Genetics and Breeding, Nanning, China

**Keywords:** direct-seeded rice, chilling stress, seed priming, germination, root morphology, enzymes activities

## Abstract

**Introduction/Background:**

Direct-seeded rice is exceptionally vulnerable to chilling stress, especially at the seed germination and seedling growth stages in the early season of the double cropping system.

**Methods:**

Therefore, we conducted two experiments to evaluate the role of various seed primings and their different concentrations of plant growth regulators [experiment 1—abscisic acid (ABA), gibberellin (GA_3_), salicylic acid (SA), brassinolide (BR), paclobutrazol, uniconazole (UN), melatonin (MT), and jasmonic acid (JA)] and osmopriming substances (chitosan, polyethylene glycol 6000 (PEG6000), and CaCl_2_) and experiment 2—GA, BR (two best), CaCl_2_ (worst), and control (CK)] on rice seedlings under low temperature condition.

**Results:**

Results showed that the maximum germination rate of 98% was recorded in GA_3_ (10 mgL^−1^) and BR (0.3 mgL^−1^) among treatments. Compared to CK, root and shoot length were improved in ABA (0.5 mgL^−1^) and GA_3_ (100 mgL^−1^) by 64% and 68%, respectively. At the same time, root and shoot weights (fresh and dry) were enhanced in Paclobutrazol (300 mgL^−1^) and GA3 among treatments. Furthermore, the average root volume, average root diameter, and total root surface area were increased by 27%, 38%, and 33% in Paclobutrazol (300 mgL^−1^), Paclobutrazol (200 mgL^−1^) and JA (1 mgL^−1^) treatments, respectively compared to CK. In the second experiment, a respective increase of 26%, 19%, 38%, and 59% was noted in SOD, POD, CAT, and APX enzyme activities in GA treatment compared to CK. Similarly, proline, soluble sugar, soluble protein, and GA content were also improved by 42%, 25.74%, 27%, and 19%, respectively, in GA treatment compared to CK. However, a respective reduction of 21% and 18% was noted in MDA and ABA content in GA treatment compared to CK. Our finding highlighted that better germination of primed-rice seedlings was associated with fresh and dry weights of the roots and shoots and the average root volume of the seedlings.

**Discussion:**

Our results suggested that GA_3_ (10 mg L^−1^) and BR (0.3 mg L^−1^) seed priming prevent rice seedlings from chilling-induced oxidative stress by regulating antioxidant enzyme activities and maintaining ABA, GA, MDA, soluble sugar, and protein content. However, further studies (transcriptome and proteome) are needed to explore the molecular mechanisms involved in seed priming-induced chilling tolerance under field conditions.

## 1 Introduction

Rice is one of the essential staple food crops globally and in China (Ali et al., 2020), and is cultivated in tropical and subtropical environments (Sun et al., 2022). In southern China, rice is cultivated twice a year where light and heat resources are generally enough, i.e., early rice and late rice. However, during germination, the spring-sowing rice is frequently affected by cold weather when the temperature declines below 12°C and lasts for more than one week, resulting in lower germination rates and reduced root growth or even complete failure of the total early-season rice (Liao et al., 2019; Liao et al., 2020). Alternatively, seed priming is an efficient, practical, and simple technique to increase rapid and uniform emergence, high seedling vigor, and yield in many field crops under unfavorable environmental conditions (Zulfiqar, 2021; Guo et al., 2022; Liu X. et al., 2022; Mohapatra et al., 2022).

Seed priming is a pre-sowing treatment that induces a physiological state more conducive to effective seed germination. Seed priming controls hydration that starts the normal metabolic process during the early stages of germination before the protrusion of the radical (Lutts et al., 2016; Johnson and Puthur, 2021). Recent studies have shown the positive effects of various seed priming methods on rice under chilling stress. For example, Nie et al. (2022) reported that seed priming increases the chilling tolerance of rice seeds during germination and growth by enhancing α-amylase activity and soluble sugar content. Furthermore, Zhu et al. (2021) showed that priming with salicylic acid (SA), gibberellin (GA_3_), CaCl_2_, and abscisic acid (ABA) improves the germination potential, germination rate, and seed vigor index of rapeseed under chilling stress, and shortens average germination time. In addition, Ur Rehman et al. (2015) also found that osmo-priming with melatonin (MT) and hormonal priming with SA are the most inexpensive treatments for improving the productivity of early planted spring maize by stimulating early seedling growth at low temperatures.

Plant growth regulators and osmotic-priming substances are the most common seed-priming agents. Plant growth regulator treatments accelerate germination and improve the stress resistance of rice seeds (Jisha et al., 2013). GA_3_, ABA, brassinolide (BR), SA, jasmonic acid (JA), MT, and paclobutrazol commonly trigger plant growth regulators. GAs are regulatory hormones that significantly improve tobacco seeds’ germination rate and seedling growth under low-temperature stress (Kan et al., 2014). Similarly, ABA is an endogenous plant hormone and plays a vital role in regulating various physiological processes in plants. For example, the development and growth of somatic embryos and seed formation affects seed dormancy (Sumera et al., 2012), reduce drought problems, and improve wheat seeds’ stress resistance (Fujita et al., 2006). Paclobutrazol and uniconazole (UN) are transported to the active site, where they effectively regulate the growth of crops at very low concentrations (Liu H. et al., 2022).

Moreover, BR plays a unique role in plant development, is widely present in the plant kingdom, and has strong biological activity at low concentrations. SA is a ubiquitous phenolic compound in plants and a product of the phenylalanine metabolic pathway (Ali et al., 2007; Laura et al., 2019). Exogenous SA maintains oxygen-producing activity and accelerates reactive oxygen species scavenging to improve the cold resistance of rice seedlings (Mutlu et al., 2013; Wang and Li, 2006). Previous studies by Khan et al. (2015) and Fahad et al. (2016) have shown that SA performs well under low and high temperatures and significantly improves the stress resistance of rice. Similarly, JA is a signaling molecule derived from fatty acids and a growth regulator that resists adverse environmental effects (Sumera et al., 2012). MT, also known as the pineal hormone, is widely found in plants (Arnao and Hernández-Ruiz, 2014). Applying MT improves the cold resistance of rice by regulating enzyme activity and the expression of cold tolerance genes (Kang et al., 2010).

Osmopriming pretreatments involve immersing seeds in an aerated solution with a low water potential and controlling the osmotic pressure by changing the concentration of the liquid, thereby preventing water absorption by the seeds (Mirmazloum et al., 2020). The osmopriming method is widely used in research and production due to its ease of operation and good effect (Nawaz et al., 2013). Commonly used initiators are organic permeable substances, such as chitosan and polyethylene glycol (PEG), and inorganic permeable substances, such as CaCl_2_. Chitosan is a polysaccharide that effectively promotes the germination of rice seeds (Orzali et al., 2017). In addition, PEG is a macromolecular substance with a significant promoting effect on seed germination (Cui et al., 2000; Chen et al., 2021). Priming with CaCl_2_ reduces the water potential and controls water absorption by seeds. The hydrolyzed ions enter the embryo cells, affect the microenvironment, and affect the growth and metabolism of the seed (Xu et al., 2009).

Owing to the scarcity of research on the beneficial effects of priming seeds under abiotic stress, little information is available on the effectiveness of various seed priming approaches to increase cold stress tolerance in rice. The objectives of the current study were to evaluate the best priming agent and optimum concentrations, (1) to improve the seed germination rate, (2) to evaluate the root and shoot growth and morphology, (3) to confirm the best seed priming *via* determination of antioxidant enzymes activities, ABA, GA and MDA contents in rice embryo under chilling stress. This study will provide the optimum plant growth regulator and osmotic priming agent concentrations for maximum direct-seeded rice seed germination and rice growth under chilling stress.

## 2 Materials and methods

### 2.1 Experimental design and treatments details

The experiment was conducted in two parts.

#### 2.1.1 Experiment 1

The Guangxi indica rice cultivar “Huanghuazhan” was selected in this experiment, which is widely used in southern China with initial germination rates of > 95% and initial seed moisture content of< 10% (on a dry weight basis). The completely randomized design (CRD) experiments were conducted in a light illumination incubator (MLR-352H-PC) at the Guangxi Academy of Agriculture Sciences, Nanning, in 2022. The seed priming treatments were (CK), the plant growth regulators [ABA (0.5, 1, 5, 10, and 100 mg L^−1^), GA_3_ (5,10, 50 and 100 mg L^−1^), SA (5, 10, 50, 100 and 150 mg L^−1^), BR (0.05, 0.1, 0.15 and 0.3 mg L^−1^), UN (5, 10, 20 and 50 mg L^−1^), paclobutrazol (50, 100, 200, 300 and 400 mg L^−1^), MT (100, 300, 500 and 700 µmol L^−1^), and JA (0.5, 1.0, 1.5 and 2 mg L^−1^)], and the osmotic initiators [chitosan (0.25% and 0.5%), PEG6000 (15%, 20% and 30%), and CaCl_2_ (0.25%, 0.5%, and 0.75%)]. The treatment details are also presented in the **Supplementary File Table 1**. Fifty seeds repeated three times from each treatment were placed on separate 150 mL petri dishes with one layer of germination paper, then put in an incubator and set at a day/night temperature of 12°C/15 °C for the chilling treatment. The light intensity was 12,000 lux. Twelve days after the treatment, the temperature was adjusted to 25°C, and the seedlings were incubated for three days.

#### 2.1.2 Experiment 2

Based on our first experimental results, the second experiment was conducted with the same cultivar under the same experimental design. We selected two best-performed primings (GA and BR) and one worst-performed (CaCl_2_) and compared them with control (CK) treatment based on higher germination for the measurement of physiological and biochemical indicators. The concentrations of GA, BR, and CaCl_2_ were 10 mg L^−1^, 0.3 mg L^−1,^and 0.5%, respectively. The 2 g seed embryo with three replicates from each treatment was placed on separate 150 mL petri dishes with one layer of germination paper, then put in an incubator (temperature and light were fixed the same as experiment 1) for germination for 96 h. This experiment investigated the physiological and biochemical mechanism of seed priming for better germination under chilling stress.

### 2.2 Seed priming procedure

The initiating solution was prepared as needed, and fifty healthy seeds were used for each treatment with three replicates. To minimize contamination during priming, the seeds were surface sterilized with 1% NaOCl solution for 30 min and rinsed three times with sterile distilled water. The seed weight ratio to solution volume was 1:5 (w/v) (Hussain et al., 2016). After 12 h, the seeds were moved to mesh bags, rinsed with tap water for 3 min, drained, and dried in a blast drying oven at 25°C for 48 h so that the total weight of the rice seeds was the same as before treatment.

### 2.3 Analysis and measurement

#### 2.3.1 Root and shoot attributes

To investigate the roots and shoots fresh weights and seedlings were removed from the incubator, and the roots and shoots were separated with scissors and weighed using a digital scale. For the root morphological attributes, the fresh roots were scanned using a Microtech Scanner (model: MRS-9600TFU2L; Shanghai, China), and Rhizo Vision Explorer v 2.0.2 software was used to analyze the root volume, diameter, and surface area. The root and shoot lengths were measured using ImageJ software. For determination of dry weights, the roots and shoots were placed in separate paper envelopes and were oven dried at 75°C for 48 h and weighed.

#### 2.3.2 Germination rates, germination potential and vigor index

The germination rates were determined every day. The optimal seed priming treatment concentration was screened out according to the germination dynamics and seedling growth status. Germination potential (GP), germination rate (GR), and vigor index (VI) were recorded through equation 1, equation 2, and equation 3 respectively (Guo et al., 2022).


Equation (1) $$GP(\%)=\frac{Number\;of\;germinated\;seed\;\left(\;on\;the\;4th\;days\;\right)\;}{Total\;number\;of\;seeds\;tested}\times 100$$

Equation (2) $$GR(\%)=\frac{Number\;of\;germinated\;seed\;\left(on\;the\;9th\;days\right)}{Total\;number\;of\;seeds\;tested}\times 100$$

$GI=\sum^{}\frac{Gt}{Dt}$ t is the GR corresponding to Dt, and Dt is the day of the germination test).


Equation (3) VI=GI×L (L is average overground length

#### 2.3.3 Antioxidant enzymes activities and non-enzymatic antioxidants

Superoxide dismutase (SOD) activity was determined by using the superoxide dismutase kit (WST-8) of Suzhou Mengxi Bio-pharmaceutical Technology Co., Ltd., expressed in U g^-1^ FW (Ukeda et al., 1999). One unit of SOD activity was the amount of extract that gave 50% inhibition in the reduction of xanthine as monitored at 450 nm. 0.5 change of A470nm per minute per gram of fresh sample in each ml of the reaction system is an enzyme activity unit. Similarly, peroxidase (POD) was determined according to the method given by Reuveni et al. (1992) and Doerge et al. (1997). Catalase (CAT) was determined according to the procedure provided by Johansson and Borg (1988). One active unit of CAT is to catalyze 1 μmol min^−1^ g^−1^ FW. The amount of mol H_2_O_2_ degraded at 240 nm μmol min^−1^ g^−1^ FW. Ascorbate peroxidase (APX) oxidizes AsA by catalyzing H_2_O_2_ and calculates the activity of APX by measuring the oxidation rate of AsA. One active unit of APX is the oxidation of 1nmolAsA per minute per 1 g of tissue at 290nm at room temperature (Ullah et al., 2016).

#### 2.3.4 Determination of free proline, malondialdehyde, total soluble sugar, and soluble protein contents

The free proline content in rice seed embryos was determined by following the procedure of Mansour and Ali (2017). Whereas, to investigate the malondialdehyde (MDA) content, the extent of the lipid peroxidation method was measured using thiobarbituric acid (TBA) by following the procedure of Spitz and Oberley (1989). Furthermore, the soluble sugar content was determined by using the anthrone method (Buysse and Merckx, 1993). Moreover, the soluble protein content was investigated using the bicinchoninic acid (BCA) method (Campion et al., 2011).

#### 2.3.5 GA_3_ and ABA contents

The HPLC method determined the ABA and GA3 contents in the rice embryo sample with minor modifications (Yang et al., 2014). Briefly, well-grinded samples of 0.2 g were over nightly incubated with 80% methanol at 4 °C. Following incubation, the samples were centrifuged at 8000×g, and the supernatant was collected. The obtained residue was further extracted for 2 h with 0.5 mL of 80% methanol and centrifuged to collect the supernatant. The collected supernatants were combined and extracted thrice with 2 mL petroleum ether at 40 °C under a nitrogen atmosphere. 1 mol L^−1^ of citric acid was, and pH was adjusted to 2–3. The samples were further extracted twice with 2 mL ethyl acetate. Ethyl acetate was dried through the blowing of nitrogen into the tube. The obtained sample was filtered and stored in a 2 mL tube. HPLC analysis was performed for hormone determination through Waters 2695 Detector.

### 2.4 Statistical analysis

Analysis of variance was used to test the seed germination rate, germination index, root and shoot attributes of rice using Statistix 8.1 software (Analytical Software, Tallahassee, FL, USA). After the assumptions were met, the data were first verified for a normal distribution. The data were analyzed in a completed randomized design using analysis of variance. Among the treatment comparisons, the least significant difference (LSD) test at p< 0.05 was used to detect differences among the means. Figures were drawn using SigmaPlot 14.0 software. R software (version 3.2) was used to conduct correlation analysis among all indicators.

## 3 Results

### 3.1 Root and shoot length

**Figure 1:**
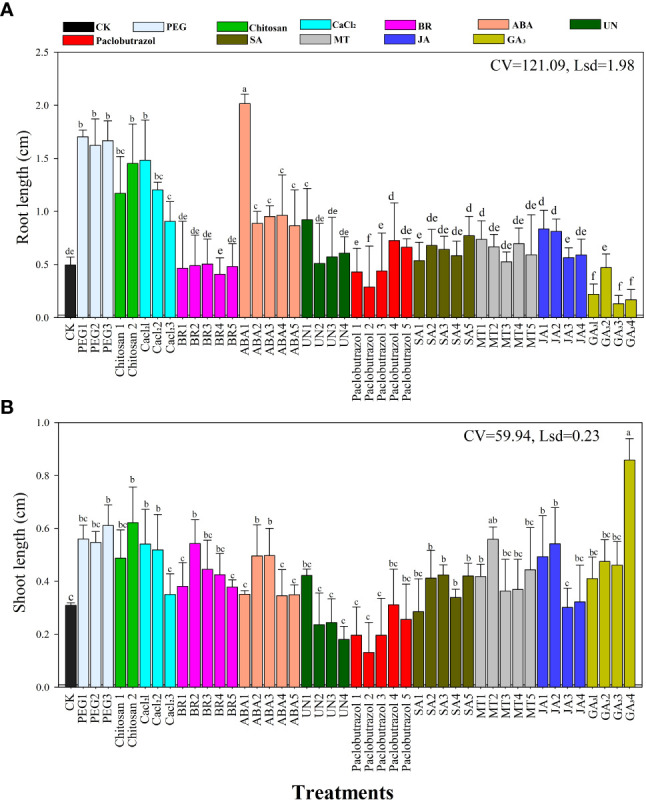
Effects of the various seed priming treatments on rice seedling **(A)** root length and **(B)** shoot lengths under chilling stress. CV—coefficient of variation; Lsd—least significant difference. Different lowercase letters indicate significant differences (P< 0.05). See **Table 1** for treatment details. Bars indicate standard errors.

The root and shoot lengths of the rice seedlings were significantly affected by the different seed priming agents and their concentrations under chilling stress as shown in **Figures 1A, B**. Compared to all other treatments, the ABA1 treatment resulted in longer roots (2.21 cm seedling^−1^) followed by PEG1 (1.7 cm seedling^−1^), PEG2 (1.6 cm seedling^−1^), and PEG3 treatments (1.62 cm seedling^−1^) (**Figure 1A**). Furthermore, the CaCl_2_ treatment improved rice root length by 1.2–1.4 mm seedling^-1^ under chilling stress. However, the SA, MT, and JA treatments slightly increased the root length but were not statistically (*P*>0.05) different from the CK.

**Table 1:** Responses of the fresh and dry weights of the roots and shoots to various seed priming treatments under different chilling stressors.

Treatments	Root fresh weight (g seedling^−1^)	Root dry weight (g seedling^−1^)	Shoot fresh weight (g seedling^−1^)	Shoot dry weight (g seedling^−1^)
CK	0.065cd	0.017b	0.113d	0.007e
PEG1	0.102b	0.015b	0.122d	0.013d
PEG2	0.127b	0.017b	0.139d	0.015d
PEG3	0.075c	0.014b	0.085de	0.011d
Chitosan1	0.110b	0.014b	0.136d	0.015d
Chitosan2	0.094bc	0.014b	0.112d	0.013d
Cacl_2_1	0.093bc	0.012bc	0.103d	0.012d
Cacl_2_2	0.066cd	0.009c	0.073e	0.007e
Cacl_2_3	0.057c	0.009c	0.156c	0.020c
BR1	0.060cd	0.009c	0.229a	0.028b
BR2	0.065d-f	0.011bc	0.192b	0.024c
BR3	0.054d-g	0.007c	0.196b	0.026b
BR4	0.076c-f	0.012bc	0.165c	0.023c
BR5	0.122bc	0.018b	0.176c	0.022c
ABA1	0.098cde	0.016b	0.220a	0.026b
ABA2	0.115bcd	0.019b	0.222a	0.027b
ABA3	0.136a	0.023a	0.166c	0.021c
ABA4	0.113bc	0.021ab	0.156c	0.020c
ABA5	0.139a	0.024a	0.207a	0.026b
UN 1	0.131a	0.014bc	0.139d	0.019cd
UN 2	0.095bcd	0.019b	0.141d	0.019cd
UN 3	0.130a	0.027a	0.139d	0.019cd
UN 4	0.104bc	0.017b	0.139d	0.017cd
Paclobutrazol1	0.067cdef	0.009c	0.070de	0.009e
Paclobutrazol 2	0.096cd	0.015b	0.119d	0.017cd
Paclobutrazol 3	0.130a	0.020b	0.153c	0.019cd
Paclobutrazol 4	0.144a	0.024a	0.138cd	0.018cd
Paclobutrazol 5	0.080cde	0.014b	0.137cd	0.019cd
SA1	0.111b	0.020ab	0.186bc	0.024c
SA2	0.122ab	0.017b	0.201b	0.023c
SA3	0.092cd	0.010c	0.141d	0.019cd
SA4	0.126ab	0.021ab	0.176b	0.023c
SA5	0.108bc	0.016b	0.171b	0.021c
MT1	0.098cde	0.017b	0.224a	0.029b
MT2	0.070def	0.014bc	0.159cd	0.021c
MT3	0.111b	0.017b	0.158cd	0.020c
MT4	0.091cd	0.015b	0.209c	0.027b
MT5	0.108b	0.019bc	0.205b	0.026b
JA1	0.120b	0.018b	0.222a	0.028b
JA2	0.083cde	0.014b	0.143d	0.018cd
JA3	0.100b	0.015b	0.151cd	0.018cd
JA4	0.045fgh	0.007	0.210b	0.029b
GA_3_1	0.068def	0.014b	0.354a	0.033a
GA_3_2	0.009i	0.002c	0.1961c	0.025b
GA_3_3	0.021gh	0.003c	0.297a	0.039a
GA_3_4	0.110b	0.017b	0.164c	0.021c
Lsd	0.0152	0.0668	0.107	ns

Lsd—Least significance difference, Different lowercase letters indicate significant differences (P< 0.05) among the different treatments within the same column. ns, non-significant.

The shoot length of the rice seedlings was affected differently by the various priming treatments under chilling stress (**Figure 1B**). Adding PEG, CaCl_2_, BR, MT, or GA_3_ priming significantly improved shoot growth compared to the control (CK). Furthermore, the GA_3_4 treatment resulted in longer shoots (0.84 cm seedling^−1^), followed by PEG3 (0.64 cm seedling^−1^), chitosan1 (0.61 cm seedling^−1^), and JA2 treatments (0.56 cm seedling^−1^) compared to the CK and the other treatments. The shortest shoots were recorded in the CK, paclobutrazol, and UN seed priming treatments.

### 3.2 Fresh and dry weight of the roots and shoots

Results showed that various seed primings significantly affected rice seedlings’ fresh roots and dry weights under chilling stress (**Table 2**). The highest root fresh weight of 0.144 g seedling^−1^ was recorded in the Paclobutrazol4 treatment compared to the control and all other treatments. Whereas seed priming with ABA5, ABA3, Paclobutrazol3, and PEG2 treatments also improved root weight up to 0.144, 0.139, 0.136, 0.130, and 0.127 g seedling^−1^ compared to CK treatment. The lowest root fresh weights of 0.065, 0.66, 0.60, and 0.57 g seedling^-1^ were recorded in the CK, BR2, BR1, and CaCl_2_3 treatments, respectively. In the case of root dry weight, UN3 resulted in the maximum root dry weight of 0.027 g seedling^-1^ followed by ABA5 (0.024 g seedling^-1^) and Paclobutrazol4 (0.024 g), whereas the lowest root dry weights of 0.007, 0.009, 0.009, 0.011, and 0.017 g were observed in the CaCl_2_, BR3, Cacl_2_3, Paclobutrazol1, and CK treatments, respectively.

**Table 2:** Changes in germination rates, germination potential, and vigor index of rice seedlings under chilling stress in response to the seed priming treatments.

Treatment	Germination Rate (%)	Germination Potential (%)	Vigor Index
CK	66.00d	3.47c	2.97d
PEG1	58.44e	0.67c	2.65d
PEG2	62.00de	2.33c	2.87d
PEG3	74.00c	3.47c	3.38cd
Chitosan1	53.11e	7.33c	2.49
Chitosan2	84.56b	12.67b	3.99c
Cacl_2_1	46.22f	2.67c	2.57d
Cacl_2_2	38.00g	0.82c	2.19d
Cacl_2_3	36.67g	0.00c	0.96e
BR1	94.00ab	9.06c	8.56ab
BR2	97.11a	7.33c	6.41bc
BR3	96.00a	9.13c	6.37b
BR4	98.00a	10.00c	8.64ab
BR5	95.11a	6.89c	5.45bc
ABA1	80.67b	5.42c	4.67c
ABA2	81.89b	6.67c	7.36bc
ABA3	80.44b	2.92c	3.42c
ABA4	55.56e	1.31c	2.35d
ABA5	65.56d	2.02c	2.86d
UN1	74.44c	6.00c	4.40bc
UN2	64.44d	2.27c	1.95d
UN3	64.89d	4.96c	2.88d
UN4	76.89c	11.33b	2.25d
Paclobutrazol1	67.78d	3.47c	1.45d
Paclobutrazol2	66.67d	2.40c	0.99e
Paclobutrazol3	55.33f	3.20c	1.30d
Paclobutrazol4	61.78de	1.33c	2.19d
Paclobutrazol5	73.33cd	4.00c	2.08d
SA1	86.22b	5.30c	3.14cd
SA2	91.11ab	6.00c	4.91c
SA3	87.33b	4.80c	4.82c
SA4	87.11b	5.50c	3.84
SA5	82.22bc	3.80c	4.18c
MT1	89.78b	10.26bc	5.28bc
MT2	96.00a	14.00b	8.28b
MT3	86.67b	9.78bc	4.51c
MT4	77.11c	1.57c	3.07
MT5	83.33cd	5.19c	4.53c
JA1	86.44c	8.53bc	5.60b
JA2	90.00b	7.33bc	5.82b
JA3	85.33b	5.12c	2.98
JA4	94.00ab	8.36bc	5.12bc
GA_3_1	95.78a	35.07a	6.33b
GA_3_2	98.67a	37.21a	13.85.a
GA_3_3	96.44a	36.67a	7.30b
GA_3_4	97.33a	34.00a	7.85b

GR, germination rate; GP, germination potential; VI, vigor index. Different lowercase letters indicate significant differences (P< 0.05) among the treatment within the same column. See **Table 1** for treatment details.

The fresh shoot weight increased the most in GA_3_1 (0.357 g), followed by GA_3_3 (0.297 g), BR1 (0.229 g), ABA2 (0.224 g), and ABA1 (0.222 g) treatments (**Table 1**). Shoot dry weight was not significantly (*P > 0.05*) affected by the different seed priming methods, as only slight increases were noted in the ABA, GA_3_, JA, and MT treatments. The lowest shoot dry weights were observed in the CK treatment. Across various concentrations, the average of the ABA treatments resulted in maximum fresh shoot weight (0.194 g) compared to the other plant growth regulators and osmo-priming treatments.

### 3.3 Root diameter and volume

**Figure 2:**
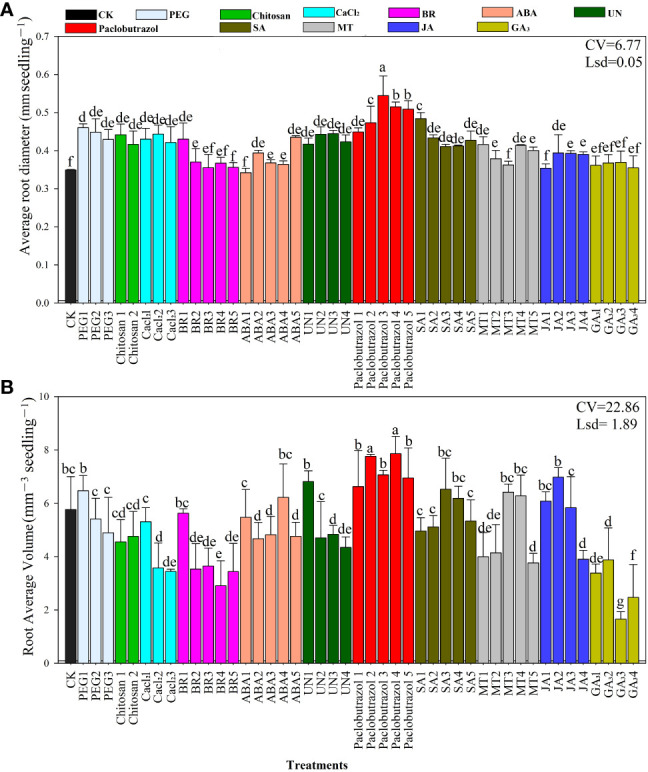
Responses of average root diameter **(A)** and root volume **(B)** to the different seed priming treatments under chilling stress. CV, coefficient of variation; Lsd, least significant difference. Different lowercase letters indicate significant differences (P< 0.05). Bar shows standard error. See **Table 1** for treatment details.

The effects of seed priming on root diameter and root volume are presented in **Figures 2A, B**. The data revealed that Paclobutrazol3, Paclobutrazol4, and Paclobutrazol5 significantly enhanced average root diameter by 0.50, 0.54, and 0.51 mm, respectively. The smallest root diameter was recorded in CK and for the average of the GA_3_ treatments (**Figure 2A**). Root volume increased in the Paclobutrazol3, Paclobutrazol4, Paclobutrazol5, and JA2 treatments with values of 7.76, 7.06, 7.86, and 6.9 mm^3^, respectively (**Figure 2B**). The smallest root volume of rice seedlings under chilling stress was recorded in the GA_3_3 treatment compared to the other treatments. The overall results show that rice seedling root diameter and volume improved in response to the paclobutrazol, JA, and ABA seed priming treatments.

### 3.4 Root average surface area

**Figure 3:**
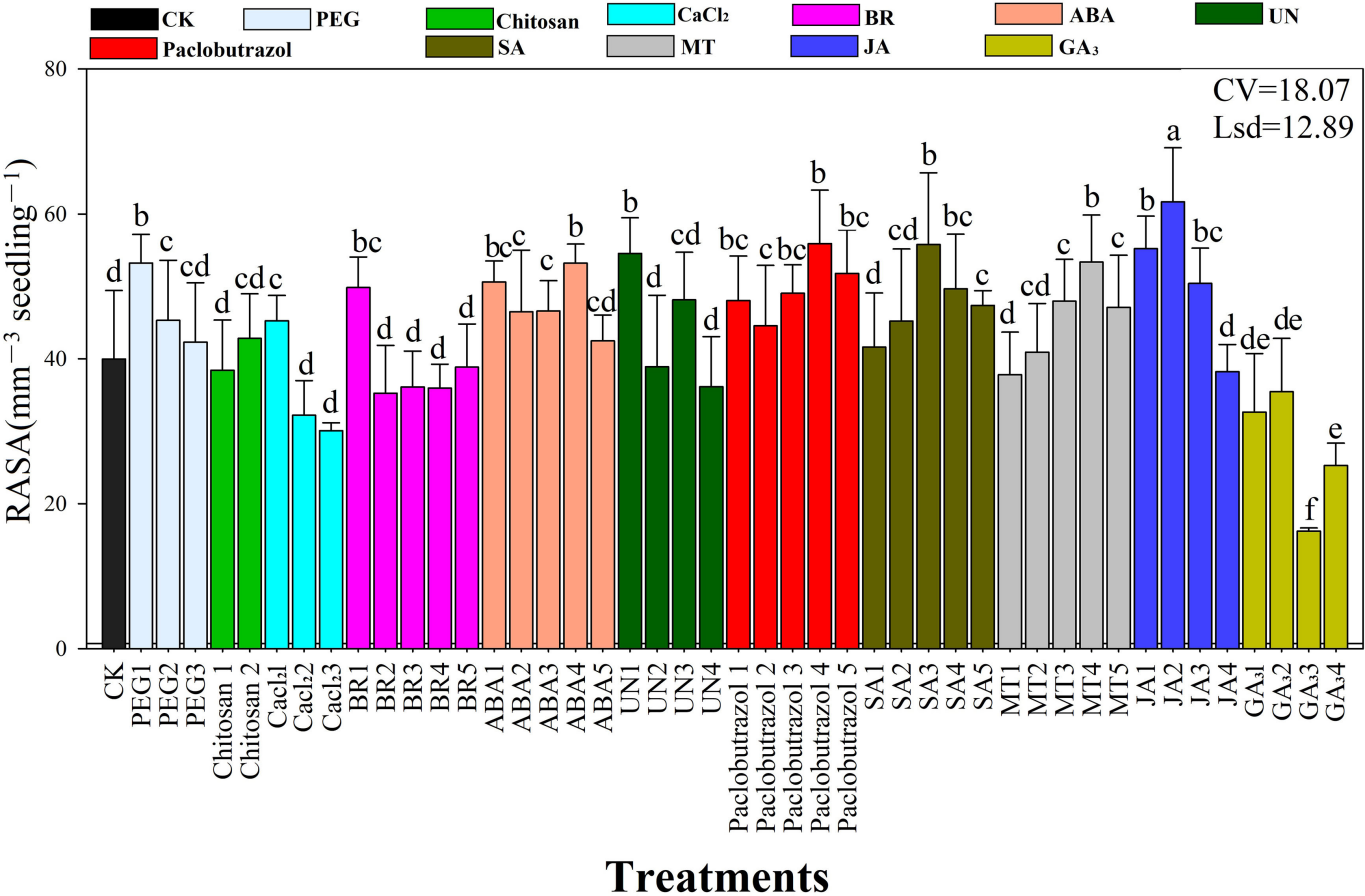
Response of root surface area to the seed priming treatments under chilling stress. RASA, root average surface area; CV, coefficient of variation; Lsd, least significant difference. Different lowercase letters indicate significant differences (P< 0.05). See **Table 1** for treatment details. Bars indicate standard errors.

The root surface area of the rice seedlings was significantly affected by the seed priming treatments under chilling stress (**Figure 3**). The results showed that seed priming with JA2 resulted in the highest (61 mm^2^) surface area, followed by MT4 (57 mm^2^), JA1 (55 mm^2^), Paclobutrazol4 (55 mm^2^), UN1 (54 mm^2^), ABA5 (53 mm^2^), PEG1 (53 mm^2^), and ABA1 (50 mm^2^). The average JA concentration increased root surface area (51.9 mm^2^), followed by paclobutrazol (53 mm^2^), while the SA rates showed maximum surface area (48.8 mm^2^) compared to the chitosan, CaCl_2_, and PEG6000 priming treatments.

### 3.5 Changes in germination rate, germination potential, and vigor index

Various seed priming under chilling stress significantly affected the rice seed germination rate, germination potential, and vigor index (**Table 1**). Results showed that the germination rate of seeds initiated by all concentrations of GA_3_ and BR was significantly increased on the 9th day and reached more than 90%, which was significantly better than other treatments, followed by SA2 and MT2. In osmopriming, the germination rate of chitosan2 reached 84.56%, which was significantly higher than other osmotic initiators.

Similarly, the germination potential of GA_3_2 and BR4 hormones and chitosan2 in osmotic initiators initiated by plant growth regulators is significantly higher than that of other initiation treatments, considerably shortening the germination time of rice seeds. In the case of seed vigor index, compared with CK, the seed vigor of rice was improved significantly by priming with GA_3_ and BR, and the priming effect of GA_3_2 and BR4 was considerably higher than that of other concentrations under the same initiator. The osmotic priming of chitosan2 significantly increased the seed vigor of rice under low temperatures. In addition, except for Paclobutrazol, all plant regulators have higher effects on seed vigor than those induced by osmotic primings.

### 3.6 Changes in antioxidant enzymes activities to various seed priming under chilling stress

**Figure 4:**
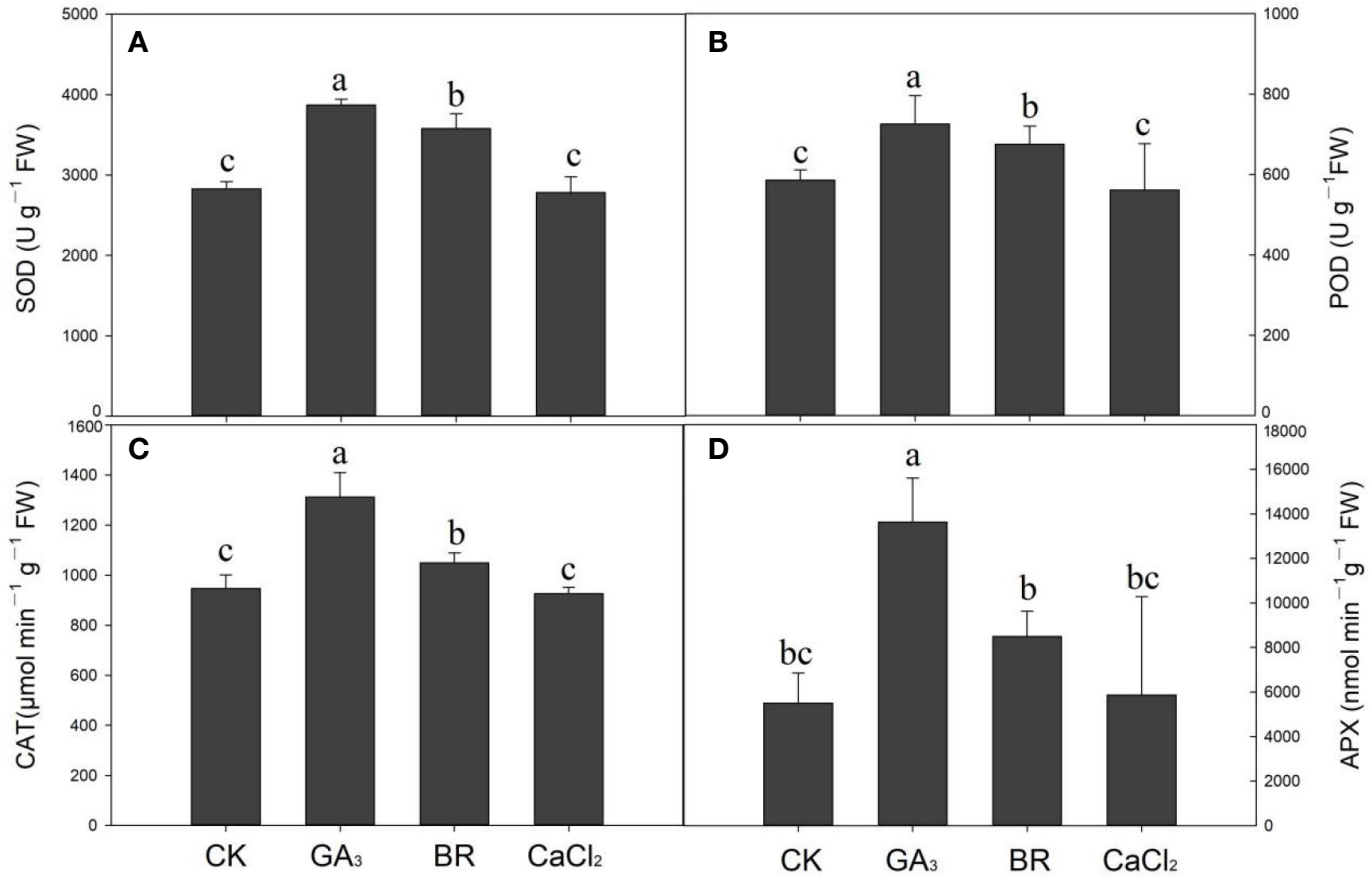
Response of antioxidant enzymes activities such as **(A)** Superoxide dismutase (SOD), **(B)** peroxidase (POD), **(C)** Catalase (CAT), and **(D)** Ascorbate peroxidase (APX) activities to various seed priming treatments under chilling stress. Lowercase letters indicate significant differences (P< 0.05) among the treatments. Bar shows standard error.

Data regarding antioxidant enzyme activities such as SOD, POD, CAT, and APX activities of rice seedlings with different seed priming under cold stress are shown in **Figure 4**. Compared to the control, SOD activities in GA and BR treatments were increased by 26.0% and 20.9%, respectively, under chilling stress (**Figure 4A** **)**. However, compared to CaCl_2_, the control treatment enhanced SOD activities by 1% but was not statistically significant. Similarly, POD activities were higher in GA and BR treatments by 19.4% and 13.3%, respectively, compared to control, while CaCl_2_ treatment reduced POD activities by 4% compared to control (**Figure 4B** **)**. Furthermore, CAT activities were higher in GA (1312.37 μmol m^−1^ g^−1^ FW) and BR (1048.84 μmol m^−1^ g^−1^ FW), followed by control (947.22 μmol m^−1^ g^−1^ FW) and CaCl_2_ (926.26 μmol m^−1^ g^−1^ FW) treatments (**Figure 4C**). Likewise, Ascorbate peroxidase (APX) activities showed the same trend, higher in GA by 59%, BR by 35%, and in CaCl_2_ by 6% as compared to control (**Figure 4D**). Overall results regarding antioxidant enzyme activities were higher in GA and BR treatments, followed by control or CaCl_2._ No statistical dissimilarity was observed between the control and CaCl2 for antioxidant enzyme activities.

### 3.7 Effect of various seed priming on MDA content under chilling stress

**Figure 5:**
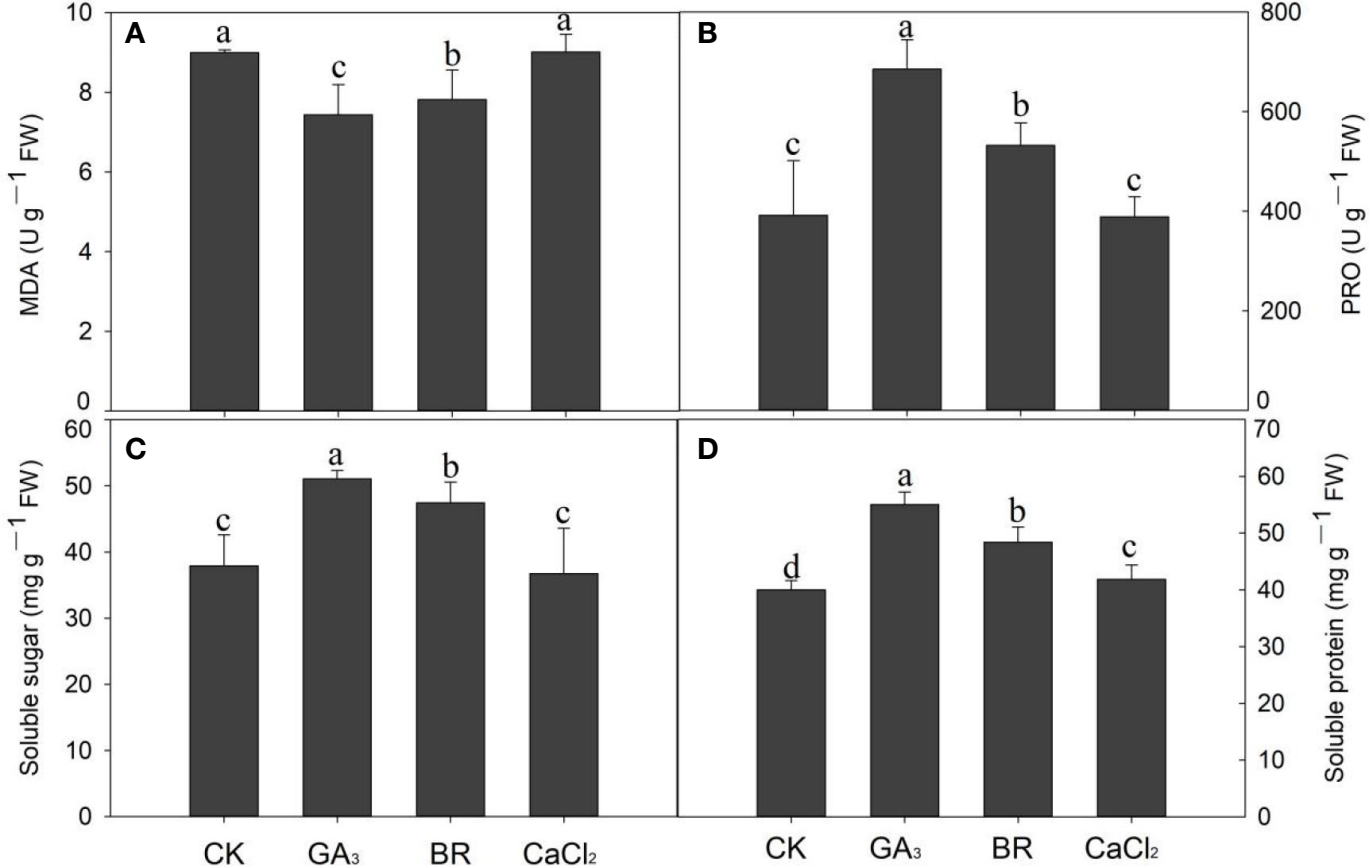
Response of **(A)** MDA, **(B)** proline, **(C)** soluble sugar, and **(D)** soluble protein content to various seed priming treatments under chilling stress. Different lowercase letters indicate significant differences (P< 0.05) among the treatments. Bar shows standard error.

Seed exposed to chilling stress with different seed priming showed that MDA content was significantly maintained by GA and BR treatments compared with control and CaCl_2_ (**Figure 5A**). The MDA contents in GA and BR treatments were significantly reduced by 21% and 15% compared to CaCl_2_. Compared to the control, it was reduced by 20% and 15% in BR and GA treatments, respectively. The rice seedling primed with GA priming was more effective in reducing MDA content than all other seed priming treatments.

### 3.8 Effect of various seed priming on proline, soluble sugar, and soluble protein under chilling stress of rice seedlings

Data regarding proline content in rice seedlings with various seed priming under chilling stress are presented in **Figure 5B**. Compared to control, seed primed with GA and BR improved proline by 42.8% and 26.2% receptively (**Figure 5B**). CaCl_2_ treatment did not significantly affect the proline content of rice compared to the control. Soluble sugar contents were also increased in GA and BR by 25.74% and 20% compared to control, while control treatment was higher by 3.09% over CaCl_2_ treatment but was not statistically (P ≤ 0.05) significant (**Figure 5C**). In cases of soluble protein content, its higher content (27%) was recorded in GA, followed by BR (17%) and CaCl_2_ (4%) compared to control (**Figure 5D**). Among all treatments, proline, soluble sugar, and soluble protein were improved in GA and BR, while the lowest values were recorded in control and CaCl_2_.

### 3.9 Effect of various seed ABA and GA contents of rice seed embryos under chilling stress of rice seedlings

**Figure 6:**
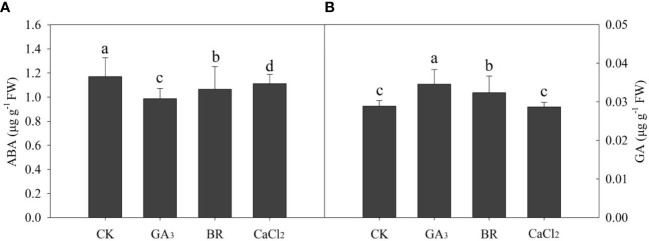
Response of **(A)** ABA and **(B)** GA_3_ content in rice embryo to different seed priming treatments under chilling stress. Different lowercase letters indicate significant differences (P< 0.05) among the treatments. Bar shows standard error.

Data on ABA and GA content in rice embryos were significantly maintained by various treatments (**Figure 6**). ABA content was decreased in the control treatment by 18%, 5%, and 7% in GA, BR, and CaCl_2,_ respectively (**Figure 6A**). The lowest ABA content was recorded in the GA-primed seeds. Whereas GA was increased by 19% and 12% in GA and BR, respectively, over CK treatment (**Figure 6B**). However, CaCl_2_ and CK treatment was not significant to each other.

### 3.10 Correlation and PCA analysis

**Figure 7:**
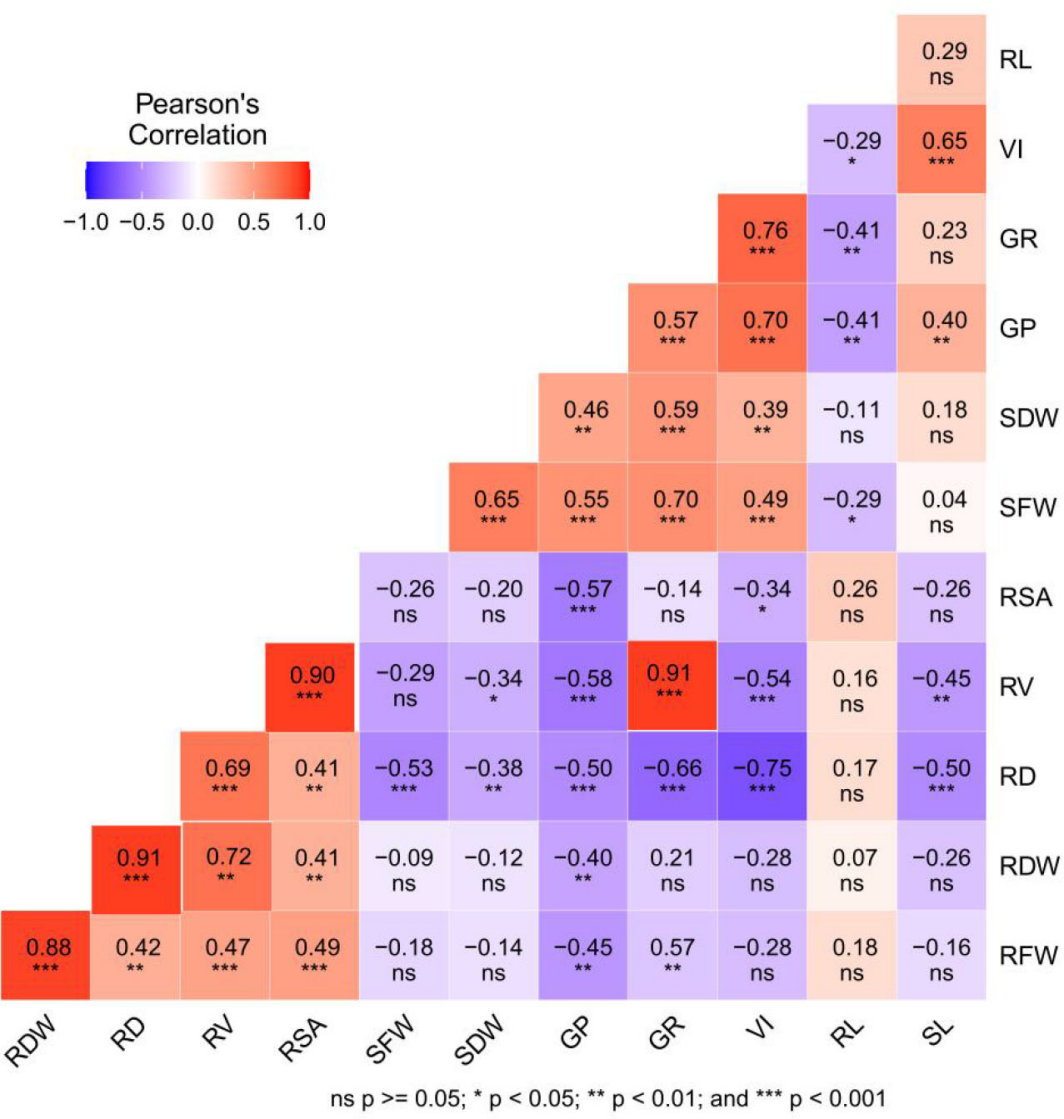
Correlation heat map among the seed germination rates and the root and shoot attributes using R software. RL, root length; SL, shoot length; RFW, root fresh weight; RDW, root dry weight; SFW, shoot fresh weight; SDW, shoot dry weight; RD, root diameter; RV, root volume; RSA, root surface area; GR, germination rate; GP, germination potential; VI, vigor index.

Correlation heat map analysis was performed to evaluate the relationships among all measured traits under chilling stress in response to the different seed priming treatments (**Figure 7**). The results show that the germination rate was positively correlated with fresh root weight (R = 0.57), shoot fresh weight (R = 0.70), shoot dry weight (R = 0.59), and average root volume (R = 0.67). Furthermore, root surface area was positively correlated with root dry weight (R = 0.59), root fresh weight (R = 0.65), and root volume (R = 0.91). Similarly, root dry weight and volume were positively correlated with fresh root weight (R = 0.90) and average root diameter (R = 0.72), respectively. However, a significant negative correlation (R = −0.50) was detected between shoot length and average root diameter. GP and VI were strongly positively correlated with SL, SFW, and SDW, while negatively correlated with root diameter.

The principle component analysis of 12 morphological indexes of rice seeds treated with different initiators under low-temperature stress was carried out, and the characteristic value and accumulative contribution rate were calculated (**Supplementary File Table 2**). Four principal components were extracted, and the cumulative contribution rate reached 79.815%. According to the contribution rate of each principal component, the germination energy, germination rate, and seed vigor were the most important factors affecting the cold tolerance of rice under different initiators.

**Figure 8:**
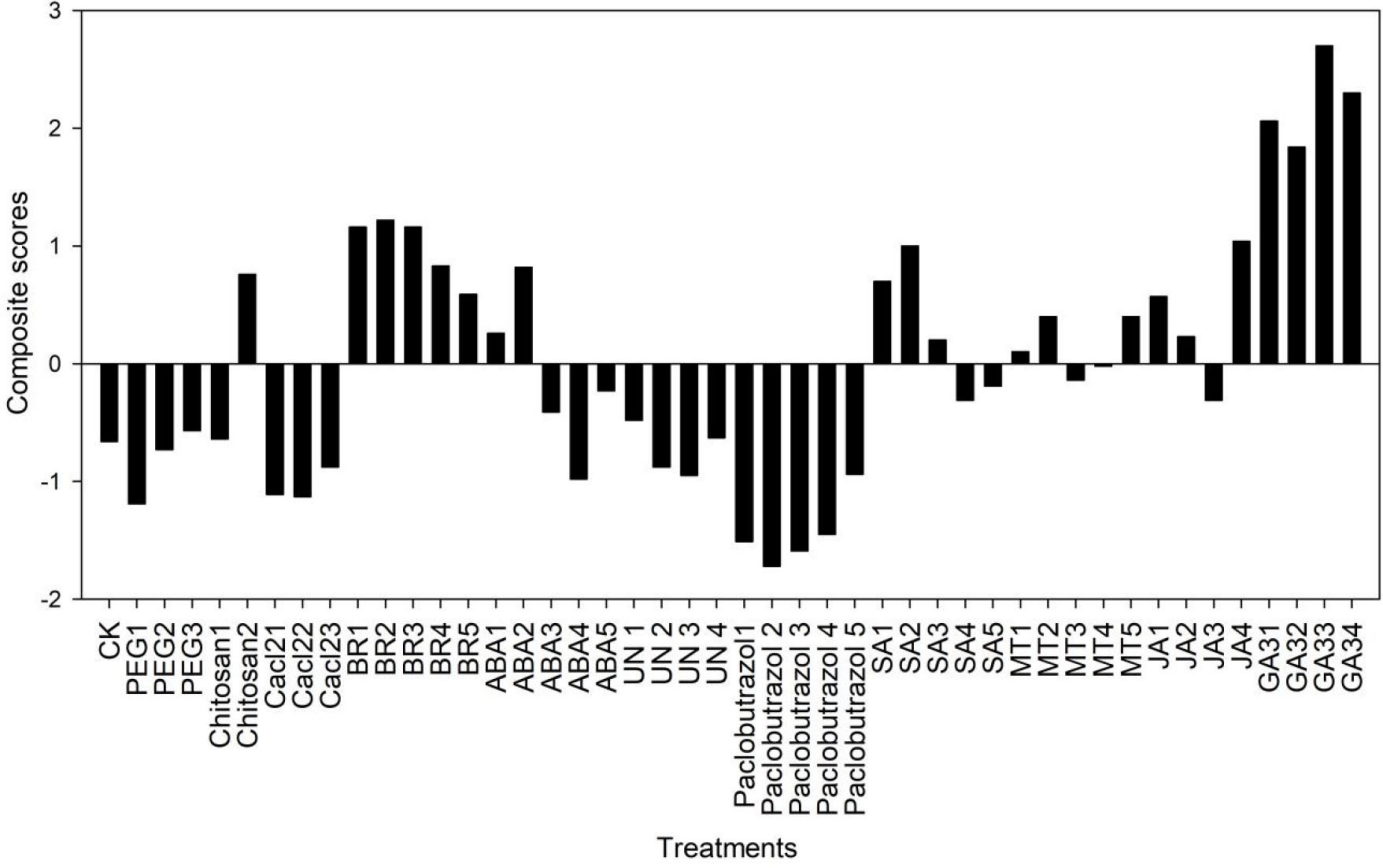
Composite scores of principal component analysis for different rice seed priming treatments at low temperatures.

The comprehensive score of principle component analysis for different rice seed priming treatments under low-temperature stress was evaluated (**Figure 8**). It was found that all concentrations of GA had the highest comprehensive scores compared with other priming treatments, followed by BR, SA(10 mg L^−1^), in which GA3 (10 mg L^−1^) and BR (0.3mg L^−1^) had the highest evaluation scores and the best priming effect. In addition, compared with other osmotic priming, 0.5% chitosan was the best priming.

## 4 Discussion

Chilling is one of the principal abiotic stressors that restrict the growth and yield of field crops. Rice is grown in tropical and subtropical climates and is extremely sensitive to chilling stress, particularly during the early seedling development and emergence stages. Direct seeding of rice is mainly used due to lower labor and production costs, as it provides a better option for seed germination and the root system. However, double-cropped early rice frequently suffers from low temperature and cold injury, which seriously affects the germination rate and establishment of the root system. The current study demonstrated the positive effects of commonly used seed priming chemicals on alleviating damage caused by cold stress.

Among the various hormone treatments, the ABA and GA_3_ treatments resulted in the longest roots and shoots, while PEG resulted in maximum root and shoot lengths compared to the CK. The possible explanation for these increases is that ABA and GA_3_ are growth promoters involved in seed germination that inhibit water uptake accompanying embryonic growth (Aziz and Pekşen, 2020). Furthermore, ABA and GA_3_ participate in the physiological and metabolic processes that initiate seed germination (Pipinis et al., 2012) and assist in reducing stress’s adverse effects (**Figure 1**). Similar to our findings, Aziz and Pekşen (2020) reported that seed priming with GA_3_ improves chickpea seedling growth under chilling stress. Furthermore, another study determined that ABA pretreatment improves photosynthetic activity, biomass and antioxidant levels in rapeseed (Zhu et al., 2021). ABA enhanced root and shoot lengths in our study.

The seedlings’ fresh and dry weights are essential for growing and initiating tillers in rice plants. In the present study, the ABA, paclobutrazol, and PEG pretreatment improved the fresh and dry weights of the roots among the various plant growth regulators and osmotic initiators compared to the CK under chilling stress. Shoot weight improved after the ABA, GA_3_, and BR pretreatment. Improvements in the fresh and dry weights of the rice seedlings under chilling stress after the ABA and GA_3_ pretreatments may be attributed to their potential role in germination’s physiological and metabolic processes (Pipinis et al., 2012). Safari et al. (2018) suggested a considerable ameliorative effect of ABA and SA pretreatments on the germinating ability of sesame seeds in a saline environment but seedling growth was retarded, which was probably a temporary response to the salinity stress. Furthermore, BR plays a vital role in regulating plant growth and development, such as cell elongation, seed germination, microtubule arrangement, cell division, and differentiation (Xiong et al., 2021). In the present study, BR improved rice seedlings’ fresh and dry weights compared to the CK (**Table 1**). In addition, the higher weight of the PEG-pretreated seedlings may be attributed to water retention. PEG priming has a noticeable effect on promoting seed germination (Pipinis et al., 2012; Muscolo et al., 2014). A positive effect of PEG pretreatment was reported on the germination of wheat seeds under drought stress (Mahpara et al., 2022), as well as in lentil genotypes (Muscolo et al., 2014), and peanut leaves and roots (Shivakrishna et al., 2018).

Rice root diameter, volume, and total root surface area are vital for regulating shoot growth and development. In the present study, the paclobutrazol, JA, ABA, and MT pretreatments improved average root diameter, root volume, and total root surface area compared to the CK. Similar to our findings, Kamran et al. (2018) and Bashir et al. (2021) reported that root diameter, root-length density, root-surface area density, and root-weight density increased in maize and okra in response to paclobutrazol, respectively. Furthermore, by controlling the physiological and biochemical responses of the plant, JA is one of the main hormones that promotes tolerance to abiotic stress (Sheteiwy et al., 2021). In contrast, ABA and MT are growth hormones involved in seed germination, inhibiting the water uptake that accompanies embryonic growth and consequently improving root morphology under drought stress (Safari et al., 2018; Zhu et al., 2021). It has been documented that ABA is extensively involved in responding to abiotic stressors, such as drought, low temperature, and osmotic stress (Fujita et al., 2006). Overall, our results show that priming seeds with various plant growth regulators, such as ABA, paclobutrazol, JA, and MT, is favorable for the root system.

The results show that the highest germination rate was found in GA_3_2 and BR4 hormones and chitosan2 osmotic priming treatments. The possible explanation for these increases is that GA and BR hormones are growth-promoting hormones involved in seed germination and inhibiting the water uptake accompanying embryonic growth (Aziz and Pekşen, 2020; Heshmati et al., 2021; Guo et al., 2022). The vigor index is the percentage of viable seeds, and vigor testing assesses the seed’s capacity to grow healthy seedlings under less-than-ideal or challenging conditions. In the current study, GA_3_2 resulted in a maximum vigor index compared to all other treatments. Similar to our findings, Ma et al. (2018) reported that GA_3_ seed priming improves seed germination rates up to 14 to 27% of perennial grass. Furthermore, Jiang et al. (2020) reported that *Isatis indigotica* fort seedlings exposed to salt stress, and seed priming treatments with GA_3_ increased the activity of antioxidant enzymes such as superoxide dismutase (SOD), peroxidase (POD), and catalase (CAT), reducing the oxidative damage induced by salt. While exposed to cold stress of Borage seed with GA_3_ seed priming at 200 mg L^−1^ gibberellic acid and 15°C, the greatest seed germination was recorded (Hasanvand et al., 2021). Furthermore, the exogenous administration of BR can successfully mitigate the harm brought on by a low temperature in maize, preserve the typical traits of seedlings in chilling stress, and assure the development and growth of plant tissue in the later stages (Sun et al., 2020), which support our results for the reducing of chilling stress in rice seed germination. Similarity for the better seed germination in chitosan2 is attributed to the fact that the energy of germination, germination percentage, lipase activity, and levels of gibberellic acid (GA_3_) and indole acetic acid (IAA) in peanuts were all raised by chitosan (Guan et al., 2009). In contrast, Adhikari et al. (2022) reported that lettuce seed hydropriming performed better compared to hormone priming under salt stress for plant growth and development. Overall our results indicated that the various plant growth regulator priming treatments improved seed germination rates from 90–96% compared to the osmopriming and control treatments.

Plants might scavenge ROS and tolerate the chilling stress due to the effective increase of antioxidant enzyme activities. In many crop plants, the protective role of SOD, POD, CAT, and APX against chilling-induced oxidative stress is visible. In the current study, seed priming treatments except for CaCl_2_ improved activities of SOD, POD, CAT, and APX, which correspond to better growth, which might be due to their better resistance to chilling stress and improved ROS scavenging ability. However, lower antioxidant activity in unprimed rice seedlings revealed that these seedlings were not capable of resisting oxidative damage caused by stress. A previous study showed that SOD plays a vital role in initiating the dismutation of superoxide, while POD and CAT modify the accumulation of H_2_O_2_ (Anjum et al., 2015; Fahad et al., 2015). Similar to our findings, Khaliq et al. (2015) and Zheng et al. (2016) reported that seed priming could enhance rice seedlings’ SOD, POD, and CAT activities. Our result confirmed that improvement in germination, root attributes, and growth of rice seedlings in GA and BR treatments were due to improvement in SOD, POD, CAT, and APX activities under chilling stress.

Seeds exposed to chilling stress under different seed priming led to higher MDA content. MDA is a lipid peroxidation biomarker and sheds light on oxidative membrane damage. Hussain et al. (2016) documented that plants exposed to unfavorable environments resulted in higher lipid peroxidation levels due to reactive oxygen species (ROS) production. In the present study, the reduction of MDA concentration in various priming treatments seedlings suggests that seed priming successfully reduced the oxidative stress caused by chilling as well as seedling damage and chilling injury. The similar positive effects of rice seed priming in averting lipid peroxidation and ROS generation was also documented by Zheng et al. (2016) and Hussain et al. (2016).

Proline content was significantly enhanced in GA_3_ and BR treatments compared to control and CaCl_2_ treatments. In plants experiencing freezing stress, the tiny molecule proline is known to serve as both an osmotic agent and a radical scavenger (Hayat et al., 2012). An increase in proline concentration mitigates the stress damage to plant cells by lowering the water potential and the plant’s osmotic adjustment (Hussain et al., 2016) and stimulating a-amylase at unfavorable temperatures. Thus we observed that GA and BR treatments increased the cold stress tolerance of rice seedlings by improving proline content in seedlings. Seed priming with GA_3_ and BR successfully mitigated the negative impacts of chilling stress in the current study. The capacity of plants to convert starch into soluble sugars is a good indicator of how well they can thrive and grow in various environments (Liu Y. et al., 2022). Prior to this, Hussain et al. (2016) revealed that improved starch metabolism was concurrent with superior germination and seedling growth performance of primed rice seedlings.

ABA and GA are plants–stress hormones and the most significant signaling molecules in plants, regulating various developmental processes and adaptive stress responses (Ye et al., 2012). In the present study, seeds exposed to chilling stress, GA and BR seed priming decreased ABA and increased GA content compared to control and CaCl_2_ treatments. It is reported that the balance of ABA controlled the cold-triggered energy absorption, trapping, and dissipation, hence reducing the excessive energy generation (Huang et al., 2017). ABA is important in forming and maintaining seed dormancy; GA promotes seed germination. GA impacts in two ways: first, by enhancing the embryo’s capacity for growth, and second, by stimulating hydrolytic enzymes. Thus our study showed that seed priming maintained the GA and ABA content in seed embryos to improve seed germination under chilling stress.

The correlations among the root attributes, dry weights, and seed germination rates revealed that the seed germination rate significantly affected root and shoot growth (**Figure 7**). This finding indicates that slow seed germination can affect plant roots and shoot growth under cold stress. Therefore, seed priming with various plant growth regulators and osmotic initiators might be suitable to improve seed germination rate, resulting in healthy plant roots and shoot growth under cold stress. PCA analysis showed that the germination energy, germination rate, and seed vigor were the most important factors affecting the cold tolerance of rice under different initiators (**Supplementary File Table 2**). The comprehensive score of principle component analysis showed that GA3 (10 mg L^−1^) and BR (0.3mg L^−1^) had the highest evaluation scores and the best priming effect, followed by SA (10 mg L^−1^) (**Figure 8**).

## 5 Conclusion

Various seed primings significantly influenced rice seedlings’ growth and germination rates under chilling stress. Rapid seed germination and strong seedling growth of rice under cold stress were observed in all seed priming regimens compared to the CK except CaCl_2_ and ABA. Especially, GA(10 mg L^−1^) and BR (0.3 mg L^−1^) seed priming enhanced increased seed germination rate by improving antioxidant enzymes activities, proline, soluble sugar, and soluble protein contents in rice embryo, and reduced MDA content, thus decreasing the damage causing by cold stress. Furthermore, the better germination rate of the priming treatments was positively correlated with the fresh and dry weights of the roots and shoots and the average root volume of the seedlings. Our findings suggested that seed priming with GA (10 mg L^−1^) and BR (0.3 mg L^−1^) is promising for improving rice seed germination and seedling growth under chilling stress. However, further studies (at the transcriptome and proteome levels) are needed to explore the mechanisms of seed priming-induced chilling tolerance under field conditions.

## Data availability statement

The raw data supporting the conclusions of this article will be made available by the authors, without undue reservation.

## Author contributions

TL and LJ designed this research. HZ, XZ, IA, XW, and MT carried out the experiments. HZ, XZ, GG, LC, IA, and XW analyzed the data and wrote the manuscript. All authors contributed to the article and approved the submitted version.

## References

[B1] Adhikari B. Olorunwa O. J. Casey Barickman J. (2022). Seed priming enhances seed germination and morphological traits of lactuca sativa l. under salt stress. Seeds 1 (2), 74–86. doi: 10.3390/seeds1020007

[B2] Ali M. B. Hahn E. J. Paek K. Y. (2007). Methyl jasmonate and salicylic acid induced oxidative stress and accumulation of phenolics in panax ginseng bioreactor root suspension cultures. Molecules 12 (3), 607–621. doi: 10.3390/12030607 17851415 PMC6149333

[B3] Ali I. He L. Ullah S. Quan Z. Wei S. Iqbal A.. (2020). Biochar addition coupled with nitrogen fertilization impacts on soil quality, crop productivity, and nitrogen uptake under double-cropping system. Food Energy Secur. 9 (3), e208. doi: 10.1002/fes3.208

[B4] Anjum S. A. Tanveer M. Hussain S. Bao M. Wang L. Khan I.. (2015). Cadmium toxicity in maize (Zea mays l.): consequences on antioxidative systems, reactive oxygen species and cadmium accumulation. Environ. Sci. pollut. Res. 22, 17022–17030. doi: 10.1007/s11356-015-4882-z 26122572

[B5] Arnao M. B. Hernández-Ruiz J. (2014). Melatonin: Plant growth regulator and/or biostimulator during stress? Trends Plant Sci. 19 (12), 789–797. doi: 10.1016/j.tplants.2014.07.006 25156541

[B6] Aziz T. Pekşen E. (2020). Seed priming with gibberellic acid rescues chickpea (Cicer arietinum l,) from chilling stress. Acta Physiol. Plantarum. 42 (8), 1–10. doi: 10.1007/s11738-020-03124-x

[B7] Bashir R. Hussain I. Rasheed R. Anwar S. Awais M. Hassan S. (2021). Seed Invigoration with Paclobutrazol Improves Seedling Growth, Physiological, Biochemical Attributes, and Fruit Yield in Okra. International Journal of Agriculture & Biology. 26 (2), 2021. doi: 10.17957/IJAB/15.1835

[B8] Buysse J. A. N. Merckx R. (1993). An improved colorimetric method to quantify sugar content of plant tissue. J. Exp. Bot. 44 (10), 1627–1629. doi: 10.1093/jxb/44.10.1627

[B9] Campion E. M. Loughran S. T. Walls D. (2011). Protein quantitation and analysis of purity. In: Walls D. Loughran S. (eds) Protein Chromatogr.: Methods Protoc. Humana Press. 681, 225–255. doi: 10.1007/978-1-60761-913-0_13

[B10] Chen X. Zhang R. Xing Y. Jiang B. Li B. Xu X.. (2021). The efficacy of different seed priming agents for promoting sorghum germination under salt stress. PloS One 16 (1), e0245505. doi: 10.1371/journal.pone.0245505 33465130 PMC7815140

[B11] Cui X. Wang X. Du H. (2000). Research progress of PEG initiated seeds. J. Tarim Agric. Reclamation Univ. 04), 47–52. Available at: http://hdl.handle.net/2097/4379.

[B12] Doerge D. R. Divi R. L. Churchwell M. I. (1997). Identification of the colored guaiacol oxidation product produced by peroxidases. Analytical Biochem. 250 (1), pp.10–pp.17. doi: 10.1006/abio.1997.2191 9234893

[B13] Fahad S. Hussain S. Saud S. Hassan S. Ihsan Z. Shah A. N.. (2016). Exogenously applied plant growth regulators enhance the morpho-physiological growth and yield of rice under high temperature. Front. Plant Sci. 202(2), 139–150. doi: 10.1111/jac.12148 PMC5003834 27625658

[B14] Fahad S. Hussain S. Saud S. Khan F. Hassan S. Nasim W.. (2015). Exogenously applied plant growth regulators affect heat-stressed rice pollens. J. Agron. Crop Sci. 202(2), 139–150. doi: 10.1111/jac.12148

[B15] Fujita M. Fujita Y. Noutoshi Y. Takahashi F. Narusaka Y. Yamaguchi-Shinozaki K.. (2006). Crosstalk between abiotic and biotic stress responses: A current view from the points of convergence in the stress signaling networks. Curr. Opin. Plant Biol. 9 (4), 436–442. doi: 10.1016/j.pbi.2006.05.014 16759898

[B16] Guan Y. J. Hu J. Wang X. J. Shao C. X. (2009). Seed priming with chitosan improves maize germination and seedling growth in relation to physiological changes under low temperature stress. J. Zhejiang Univ. Sci. B 10 (6), 427–433. doi: 10.1631/jzus.B0820373 19489108 PMC2689555

[B17] Guo Y. Li D. Liu L. Sun H. Zhu L. Zhang K.. (2022). Seed priming with melatonin promotes seed germination and seedling growth of triticale hexaploide l, under PEG-6000 induced drought stress. Front. Plant Sci. 13. doi: 10.3389/fpls.2022.932912 PMC9280350 35845711

[B18] Hasanvand H. Parmoon G. Moosavi S. A. Siadat S. A. (2021). Effects of seed priming with gibberellic acid on cardinal temperatures of borage (Borage officinalis l,) seed germination. Iranian J. Seed Sci. Technol. 10 (3), 43–56. doi: 10.22092/IJSST.2020.343157.1347

[B19] Hayat S. Hayat Q. Alyemeni M. N. Wani A. S. Pichtel J. Ahmad A. (2012). Role of proline under changing environments: a review. Plant Signaling Behav. 7 (11), 1456–1466. doi: 10.4161/psb.21949 PMC3548871 22951402

[B20] Heshmati S. Dehaghi M. A. Farooq M. Wojtyla Ł. Maleki K. Heshmati S. (2021). Role of melatonin seed priming on antioxidant enzymes and biochemical responses of carthamus tinctorius l, under drought stress conditions. Plant Stress 2, 100023. doi: 10.1016/j.stress.2021.100023

[B21] Huang X. Shi H. Hu Z. Liu A. Amombo E. Chen L.. (2017). ABA is involved in regulation of cold stress response in bermudagrass. Front. Plant Sci. 8, 1613. doi: 10.3389/fpls.2017.01613 29081782 PMC5645512

[B22] Hussain S. Khan F. Hussain H. A. Nie L. (2016). Physiological and biochemical mechanisms of seed priming-induced chilling tolerance in rice cultivars. Front. Plant sci. 7, 116. doi: 10.3389/fpls.2016.00116 26904078 PMC4746480

[B23] Jiang X. W. Zhang C. R. Wang W. H. Xu G. H. Zhang H. Y. (2020). Seed priming improves seed germination and seedling growth of isatis indigotica fort, under salt stress. HortScience 55 (5), 647–650. doi: 10.21273/HORTSCI14854-20

[B24] Jisha K. Vijayakumari K. Puthur J. T. (2013). Seed priming for abiotic stress tolerance: an overview. Acta Physiol. Plantarum 35 (5), 1381–1396. doi: 10.1007/s11738-012-1186-5

[B25] Johansson L. H. Borg L. H. (1988). A spectrophotometric method for determination of catalase activity in small tissue samples. Analytical Biochem. 174 (1), 331–336. doi: 10.1016/0003-2697(88)90554-4 3064653

[B26] Johnson R. Puthur J. T. (2021). Seed priming as a cost effective technique for developing plants with cross tolerance to salinity stress. Plant physiology and biochemistry 162, 247–257. doi: 10.1016/j.plaphy.2021.02.034 33711718

[B27] Kamran M. Cui W. Ahmad I. Meng X. Zhang X. Su W.. (2018). Effect of paclobutrazol, a potential growth regulator on stalk mechanical strength, lignin accumulation and its relation with lodging resistance of maize. Plant Growth Regul. 84, 317–332. doi: 10.1007/s10725-017-0342-8

[B28] Kan P. Wang S. S. Ma W. G. Long Z. L. Yang H. Q. Song B. P.. (2014). Study on enhancement of chilling and drought resistance in tobacco seeds and seedlings by seed priming with gibberellin. Seed 02, 30–34+38. Available at: https://www.cabdirect.org/cabdirect/abstract/20143376516.

[B29] Kang K. Lee K. Park S. Kim Y. S. Back K. (2010). Enhanced production of melatonin by ectopic overexpression of human serotonin n-acetyltransferase plays a role in cold resistance in transgenic rice seedlings. J. pineal. Res. 49 (2), 176–182. doi: 10.1111/j.1600-079X.2010.00783.x 20586889

[B30] Khaliq A. Aslam F. Matloob A. Hussain S. Geng M. Wahid A.. (2015). Seed priming with selenium: Consequences for emergence, seedling growth, and biochemical attributes of rice. Biol. Trace Elem. Res. 166, 236–244. doi: 10.1007/s12011-015-0260-4 25690516

[B31] Khan M. I. R. Fatma M. Per T. S. Anjum N. A. Khan N. A. (2015). Salicylic acid-induced abiotic stress tolerance and underlying mechanisms in plants. Front. Plant Sci. 6, 462. doi: 10.3389/fpls.2015.00462 26175738 PMC4485163

[B32] Laura A. Moreno-Escamilla J. O. Rodrigo-García J. Alvarez-Parrilla E. (2019). “Postharvest physiology and biochemistry of fruits and vegetables. Phenolic Compounds, Woodhead Publishing, Queretaro, 253e271. doi: 10.1016/B978-0-12-813278-4.00012-9

[B33] Liao X. Huang M. Yong Y. Li Y. Zhou S. Qin C. (2020). Research progress and prospect of the impact of climate change on agriculture in guangxi. Meteorol. Res. Appl. 41 (04), 72–80.

[B34] Liao X. Li Y. Huang M. Shi C. Qin Z. Huang Y. (2019). Evolution characteristics of chilling injury of double cropping rice in guangxi from 1961 to 2016. Meteorol. Res. Appl. 40 (04), 41–45+112.

[B35] Liu Y. Qi Z. Wei J. Yu J. Xia X. (2022). Brassinosteroids promote starch synthesis and the implication in low-light stress tolerance in solanum lycopersicum. Environ. Exp. Bot. 201, 104990. doi: 10.1016/j.envexpbot.2022.104990

[B36] Liu X. Quan W. Bartels D. (2022). Stress memory responses and seed priming correlate with drought tolerance in plants: An overview. Planta 255 (2), 45. doi: 10.1007/s00425-022-03828-z 35066685 PMC8784359

[B37] Liu H. Xu Y. Wang Y. Liu C. Chen J. Fan S.. (2022). Study on endocrine disruption effect of paclobutrazol and uniconazole on the thyroid of male and female rats based on lipidomics. Ecotoxicol. Environ. Saf. 234, 113386. doi: 10.1016/j.ecoenv.2022.113386 35286959

[B38] Lutts S. Benincasa P. Wojtyla L. Kubala S. Pace R. Lechowska K.. (2016). “Seed priming: new comprehensive approaches for an old empirical technique,” in New challenges in seed biology - basic and translational research driving seed technology, 1–46.

[B39] Ma H. Y. Zhao D. D. Ning Q. R. Wie J. P. Li Y. Wang M. M.. (2018). A multi-year beneficial effect of seed priming with gibberellic acid-3 (GA3) on plant growth and production in a perennial grass, leymus chinensis. Sci. Rep. 8, 13214. doi: 10.1038/s41598-018-31471-w 30181574 PMC6123402

[B40] Mahpara S. Zainab A. Ullah R. Kausar S. Bilal M. Latif M. I.. (2022). The impact of PEG-induced drought stress on seed germination and seedling growth of different bread wheat (Triticum aestivum l,) genotypes. PloS One 17 (2), e0262937. doi: 10.1371/journal.pone.0262937 35148345 PMC8836350

[B41] Mansour M. M. F. Ali E. F. (2017). Evaluation of proline functions in saline conditions. Phytochemistry 140, 52–68. doi: 10.1016/j.phytochem.2017.04.016 28458142

[B42] Mirmazloum I. Kiss A. Erdélyi É. Ladányi M. Németh É.Z. Radácsi P. (2020). The effect of osmopriming on seed germination and early seedling characteristics of carum carvi l. Agriculture 10 (4), 94. doi: 10.3390/agriculture10040094

[B43] Mohapatra S. Sirhindi G. Dogra V. (2022). Seed priming with brassinolides improves growth and reinforces antioxidative defenses under normal and heat stress conditions in seedlings of brassica juncea. Physiol. Plantarum 174 (6), e13814. doi: 10.1111/ppl.13814 36326060

[B44] Muscolo A. Sidari M. Anastasi U. Santonoceto C. Maggio A. (2014). Effect of PEG-induced drought stress on seed germination of four lentil genotypes. J. Plant Interact. 9 (1), 354–363. doi: 10.1080/17429145.2013.835880

[B45] Mutlu S. Karadağoğlu Ö. Atici Ö. Nalbantoğlu B. (2013). Protective role of salicylic acid applied before cold stress on antioxidative system and protein patterns in barley apoplast. Biol. Plantarum 57 (3), 507–513. doi: 10.1007/s10535-013-0322-4

[B46] Nawaz J. Hussain M. Jabbar A. Nadeem G. A. Sajid M. Subtain M. U.. (2013). Seed priming a technique. Int. J. Agric. Crop Sci. 6 (20), 1373. Available at: https://www.cabdirect.org/cabdirect/abstract/20143069352.

[B47] Nie L. Song S. Yin Q. Zhao T. Liu H. He A.. (2022). Enhancement in seed priming-induced starch degradation of rice seed under chilling stress *via* GA-mediated α-amylase expression. Rice 15 (1), 1–13. doi: 10.1186/s12284-022-00567-3 35344097 PMC8960536

[B48] Orzali L. Corsi B. Forni C. Riccioni L. (2017). Chitosan in Agriculture: A New Challenge for Managing Plant Disease. In Shalaby E. A. (a cura di), Biological Activities and Application of Marine Polysaccharides (pp. 17–36). INTECH. doi: 10.5772/66840

[B49] Pipinis E. Milios E. Kiamos N. Mavrokordopoulou O. Smiris P. (2012). Effects of stratification and pre-treatment with gibberellic acid on seed germination of two carpinus species. Seed Sci. Technol. 40 (1), 21–31. doi: 10.15258/sst.2012.40.1.03

[B50] Reuveni R. Shimoni M. Karchi Z. Kuc J. (1992). Peroxidase activity as a biochemical marker for resistance of muskmelon(Cucumis melo) to pseudoperonospora cubensis. Phytopathology 82 (7), 749–753. doi: 10.1094/Phyto-82-749

[B51] Safari H. Hosseini S. M. Azari A. Rafsanjani M. H. (2018). Effects of seed priming with ABA and SA on seed germination and seedling growth of sesame (Sesamum indicum l,) under saline conditions. Aust. J. Crop Sci. 12 (9), 1385–1392. doi: 10.21475/ajcs.18.12.09.PNE940

[B52] Sheteiwy M. S. Shao H. Qi W. Daly P. Sharma A. Shaghaleh H.. (2021). Seed priming and foliar application with jasmonic acid enhance salinity stress tolerance of soybean (Glycine max l,) seedlings. J. Sci. Food Agric. 101 (5), 2027–2041. doi: 10.1002/jsfa.10822 32949013

[B53] Shivakrishna P. Reddy K. A. Rao D. M. (2018). Effect of PEG-6000 imposed drought stress on RNA content relative water content (RWC) and chlorophyll content in peanut leaves and roots. Saudi J. Biol. Sci. 25 (2), 285–289. doi: 10.1016/j.sjbs.2017.04.008 29472779 PMC5815994

[B54] Spitz D. R. Oberley L. W. (1989). An assay for superoxide dismutase activity in mammalian tissue homogenates. Analytical Biochem. 179 (1), 8–18. doi: 10.1016/0003-2697(89)90192-9 2547324

[B55] Sumera I. Asghari B. Noshin I. (2012). Abscisic acid (ABA) seed soaking induced changes in physiology of two wheat cultivars under water stress. Pakistan J. Bot. 44(Suppl 1), 51–56. Available at: https://www.pakbs.org/pjbot/PDFs/44(SI1)/08.pdf.

[B56] Sun Y. He Y. Irfan A. R. Liu X. Yu Q. Zhang Q.. (2020). Exogenous brassinolide enhances the growth and cold resistance of maize (Zea mays l,) seedlings under chilling stress. Agronomy 10 (4), 488. doi: 10.3390/agronomy10040488

[B57] Sun C. Zhang H. Ge J. Wang C. Li L. Xu L. (2022). Rice mapping in a subtropical hilly region based on sentinel-1 time series feature analysis and the dual branch BiLSTM model. Remote Sens. 14 (13), 3213. doi: 10.3390/rs14133213

[B58] Ukeda H. Kawana D. Maeda S. Sawamura M. (1999). Spectrophotometric assay for superoxide dismutase based on the reduction of highly water-soluble tetrazolium salts by xanthine-xanthine oxidase. Biosci. biotechnol. Biochem. 63 (3), 485–488. doi: 10.1271/bbb.63.485 27393255

[B59] Ullah S. Kolo Z. Egbichi I. Keyster M. Ludidi N. (2016). Nitric oxide influences glycine betaine content and ascorbate peroxidase activity in maize. South Afr. J. Bot. 105, 218–225. doi: 10.1016/j.sajb.2016.04.003

[B60] Ur Rehman H. Iqbal H. Basra S. M. Afzal I. Farooq M. Wakeel A.. (2015). Seed priming improves early seedling vigor growth and productivity of spring maize. J. Integr. Agric. 14 (9), 1745–1754. doi: 10.1016/S2095-3119(14)61000-5

[B61] Wang L. J. Li S. H. (2006). Salicylic acid-induced heat or cold tolerance in relation to Ca^2+^ homeostasis and antioxidant systems in young grape plants. Plant Sci. 170 (4), 685–694. doi: 10.1016/j.plantsci.2005.09.005

[B62] Xiong M. Chu L. Li Q. Yu J. Yang Y. Zhou P.. (2021). Brassinosteroid and gibberellin coordinate rice seed germination and embryo growth by regulating glutelin mobilization. Crop J. 9 (5), 1039–1048. doi: 10.1016/j.cj.2020.11.006

[B63] Xu F. F. Ye L. M. Wang H. Q. Xiong Z. H. (2009). CaCl_2_ soaking seeds on salt resisrance of rice seedlings. ,Henan Agric. Sci. 12), 44–45+47.

[B64] Yang R. Yang T. Zhang H. Qi Y. Xing Y. Zhang N.. (2014). Hormone profiling and transcription analysis reveal a major role of ABA in tomato salt tolerance. Plant Physiol. Bioch 77, 23–34. doi: 10.1016/j.plaphy.2014.01.015 24531233

[B65] Ye N. Jia L. Zhang J. (2012). ABA signal in rice under stress conditions. Rice 5 (1), 1–9. doi: 10.1186/1939-8433-5-1 24764501 PMC3834477

[B66] Zheng M. Tao Y. Hussain S. Jiang Q. Peng S. Huang J.. (2016). Seed priming in dry direct-seeded rice: Consequences for emergence, seedling growth and associated metabolic events under drought stress. Plant Growth Regul. 78, 167–178. doi: 10.1007/s10725-015-0083-5

[B67] Zhu Z. H. Sami A. Xu Q. Q. Wu L. L. Zheng W. Y. Chen Z. P.. (2021). Effects of seed priming treatments on the germination and development of two rapeseed (Brassica napus l,) varieties under the co-influence of low temperature and drought. PloS One 16 (9), e0257236. doi: 10.1371/journal.pone.0257236 34529689 PMC8445418

[B68] Zulfiqar F. (2021). Effect of seed priming on horticultural crops. scientia. Horticulturae 286, 110197. doi: 10.1016/j.scienta.2021.110197

