# Supplementary material for: Effects of various seed priming on morphological, physiological, and biochemical traits of rice under chilling stress
Source: Front Plant Sci. 2023 Mar 13;14:1146285. doi: 10.3389/fpls.2023.1146285 (PMC10040639; doi:10.3389/fpls.2023.1146285)
Supplement: Supplementary file 1 [file Table_1.docx]

Supplementary File

**S-Table 1.** Experimental treatment details of the plant growth regulators and osmotic

| Treatments | concentration | | | | |
| --- | --- | --- | --- | --- | --- |
|  | 1 | 2 | 3 | 4 | 5 |
| ABA | 0.5 mg·L^-1^ | 1 mg·L^-1^ | 5 mg·L^-1^ | 10 mg·L^-1^ | 100 mg·L^-1^ |
| GA_3_ | 5 mg·L^-1^ | 10 mg·L^-1^ | 50 mg·L^-1^ | 100 mg·L^-1^ | / |
| SA | 5 mg·L^-1^ | 10 mg·L^-1^ | 50 mg·L^-1^ | 100 mg·L^-1^ | 150 mg·L^-1^ |
| BR | 0.05 mg·L^-1^ | 0.1 mg·L^-1^ | 0.15mg·L^-1^ | 0.3 mg·L^-1^ | / |
| Paclobutrazol | 50 mg·L^-1^ | 100 mg·L^-1^ | 200 mg·L^-1^ | 300 mg·L^-1^ | 400 mg·L^-1^ |
| UN | 5 mg·L^-1^ | 10mg·L^-1^ | 20mg·L^-1^ | 50mg·L^-1^ | / |
| MT | 100 umol·L^-1^ | 300 umol·L^-1^ | 500 umol·L^-1^ | 700 umol·L^-1^ | / |
| JA | 0.5 mg·L^-1^ | 1 mg·L^-1^ | 1.5 mg·L^-1^ | 2 mg·L^-1^ | / |
| PEG 6000 | 15%, | 20% | 30% | / | / |
| CaCl_2_ | 0.25 % | 0.5 % | 0.75 % | / | / |
| Chitosan | 0.25% | 0.5 % | / | / | / |
| Water (CK) | CK | / | / | / | / |

**S-Table 2.** Principal component analysis of 12 morphological indicators induced by rice seeds

| Traits | PC | | | |
| --- | --- | --- | --- | --- |
|  | PC1 | PC2 | PC3 | PC4 |
| germination potential（GP） | 0.843 | 0.044 | -0.112 | -0.025 |
| vigor index（VI） | 0.832 | 0.182 | 0.336 | -0.105 |
| root diameter（RD） | -0.814 | -0.073 | -0.264 | 0.079 |
| root volume（RV） | -0.803 | 0.302 | -0.002 | 0.336 |
| germination rate（GR） | 0.711 | 0.499 | 0.067 | 0.084 |
| root surface area（RSA） | -0.663 | 0.415 | 0.256 | 0.418 |
| shoot fresh weight（SFW） | 0.642 | 0.526 | -0.115 | 0.142 |
| root fresh weight（RFW） | -0.601 | 0.488 | 0.368 | -0.351 |
| shoot dry weight（SDW） | 0.527 | 0.393 | 0.004 | 0.472 |
| root fresh weight（RDW） | -0.526 | 0.569 | 0.242 | -0.468 |
| shoot length（SL） | 0.505 | -0.329 | 0.727 | -0.032 |
| root length（RL） | -0.352 | -0.362 | 0.644 | 0.343 |
| eigenvalue | 5.362 | 1.785 | 1.415 | 1.016 |
| variance contribution rate（%） | 44.684 | 14.874 | 11.792 | 8.466 |
| total variance（%） | 44.684 | 59.558 | 71.35 | 79.815 |
